# Evaluation of two different semi-automated homogenization techniques in microbiological diagnosis of periprosthetic joint infection: disperser vs. bead milling method

**DOI:** 10.1186/s12879-022-07775-8

**Published:** 2022-10-17

**Authors:** Heime Rieber, Andre Frontzek, Stephanie Heinrich, Bertram Barden, Thomas Kortstegge, Thomas Dienstknecht, Andreas Breil-Wirth, Mathias Herwig, Jörg Jerosch, Ralf Pinkernell, Martin Ulatowski

**Affiliations:** 1 MVZ Dr. Stein and Colleagues, Division of Microbiology, Tomphecke 45, D-41169 Mönchengladbach, Germany; 2 Klinik für Orthopädie und Unfallchirurgie, Krankenhaus Düren, Düren, Germany; 3 Klinik für Orthopädie und Unfallchirurgie, Johanna-Etienne-Krankenhaus, Neuss, Germany; 4 Klinik für Orthopädie und Unfallchirurgie, Sana Krankenhaus, Radevormwald, Germany

**Keywords:** Periprosthetic joint infection, Microbiological diagnostics, Tissue homogenization techniques

## Abstract

**Background:**

In microbiological diagnosis of periprosthetic joint infection (PJI) there is no consensus regarding the most suitable and optimal number of specimens to be cultured or the most effective technique of tissue processing. This comparative study analysed the accuracy of two semi-automated homogenization methods with special focus on the volume and exact origin of each sample.

**Methods:**

We investigated a total of 722 periprosthetic tissue samples. PJI was defined according to the new scoring system for preoperative and intraoperative criteria. We compared the performance of our routinely used single tissue processing by disposable high-frequency disperser with the bead milling method.

**Results:**

Eighty patients were included. Among forty classified PJIs, 34 patients yielded positive culture results. In 23 cases (68%) exact concordant results were generated with both techniques. However, in seven cases (20%) processing by the disperser and in four cases (12%) by bead milling provided additional positive samples, but without significant difference since the major definition criteria were met in all cases. The percentage of positive results was influenced by the volume and origin of the tissue samples. Results for small tissue samples tended to be better using the bead milling method. This might lead to improved preoperative arthroscopic diagnosis, as the volume of biopsies is generally limited. Six patients had negative results due to previous antimicrobial therapy. Forty other patients were classified as aseptic failures. Neither procedure resulted in any contamination.

**Conclusion:**

Both methods enable reliable processing of tissue samples for diagnosis of PJI and are suitable for routine use.

## Introduction

Microbiological investigations play a key role in the diagnosis of periprosthetic joint infection (PJI). In contrast to many organ-related infections which cause acute symptoms, PJI often has a chronically insidious course. Depending on the joint and patient collective, these cases can account for up to 50% of the total number of infections (own data). The consequences for the patient are considerable, since almost every case sooner or later requires surgical intervention. The development of the infection is closely related to the variable growth behaviour of the pathogens. Many microorganisms are able to colonize the surface of a foreign body, creating a biofilm to protect them from their environment. If they cause infections in the tissue surrounding the devices, bacteria can survive as sessile or slow-growing variants, making diagnostics and therapy a challenge [1]. Furthermore, chronic inflammation is histologically characterized by predominant fibrous granulation tissue, while the proportion of neutrophils, the hallmark of an acute infection process, is usually very low. This places special demands on the laboratory in terms of processing and culture methods. Unfortunately, there are still no standard procedures for processing or cultivation. We have recently published data on the significance of culture media for diagnostics in PJI [2, 3].

It is undisputed that semi-automated homogenization of tissue samples is superior to any manual method [4]. However, these methods are still compared with one another in various publications. To our knowledge, this is the first study that has evaluated the performance of two different semi-automated homogenization techniques and their effect on the yield of bacteria, additionally taking into account the number, volume and origin of the samples. We compared our routine procedure in which we process single tissue samples by disposable high-frequency disperser with the bead milling method (mechanized agitation) which enables simultaneous handling of several samples.

## Materials and methods

### Study population/definition of infection

This comparative study was conducted between 2019 and 2020 and included patients from three different hospitals with which our laboratory has a cooperation agreement for microbiological diagnostics. We investigated about 800 tissue samples from a total of 90 patients, almost equally distributed among the hospitals. The patients had undergone revision arthroplasty of the hip or knee because of presumed infection or aseptic failure (AF). We based our definition of PJI on the new scoring system for preoperative and intraoperative criteria published by Parvizi et al. [5]. Two positive tissue cultures with the same microorganisms and/or the presence of a sinus tract communicating with the prosthesis were considered as major criteria for infection. The following parameters were regarded as preoperative minor criteria: elevated serum CRP (> 1 mg/dL), D-dimer (> 860 ng/mL), and erythrocyte sedimentation rate (> 30 mm/h) assigned with 2, 2 and 1 points. Furthermore, elevated synovial fluid white blood cell count (> 3000 cells/µL), alpha-defensin (signal-to-cut off ratio > 1), leucocyte esterase (++), polymorphonuclear percentage (> 80%), and synovial CRP (> 6.9 mg/L) received 3, 3, 3, 2, and 1 points, respectively. Patients with an aggregate score of greater than or equal to 6 were considered to be infected. For patients with a lower score, intraoperative findings of positive histology, purulence, and a single positive culture were included and assigned 3, 3, and 2 points. Combined with the preoperative score, a total of greater than or equal to 6 was considered infected, a final score between 4 and 5 was inconclusive, and a score of 3 or less was considered not infected. Histopathological analysis was interpreted according to the classification by Krenn et al. [6].

### Ethics

The study was approved by the ethics committee of the General Medical Council of North-Rhine, Düsseldorf, Germany. All patients gave their consent to participate in this study.

### Specimen processing

A minimum set of four tissue samples per patient and method was a prerequisite for participation in the study. The specimens were taken from the neo-synovium, the area around the acetabulum and diverse suspicious sites in the periprosthetic membrane. On the basis of previous experience, samples ≥ 1 cm^3^ in size, corresponding to a weight of ≥ 1.5 g, were required, provided that the operating processes allowed this. Each sample was taken with a separate, sterile instrument. All samples were collected in the operating room using different transport vials depending on the method. For routine diagnostics, each sample was transferred to an individually packaged, sterile 25 ml tube (Sarstedt, Australia) with a screw top, and for the bead milling method a 15 ml tube filled with 50 ceramic beads of 2.8/5.0 mm provided by the supplier (Bertin Technologies, USA) was used. This was individually packaged and sterilized by steam. Sterilization was controlled and documented with process indicators as well as bioindicators. To prevent tissue samples drying up, each vial was covered (3–5 ml depending on the size) in the operating room with single-use, separate, sterile 0.9% sodium chloride solution. All samples were transferred to the laboratory within four hours.

In the laboratory the 25 ml tubes were directly homogenized at a laminar air flow bench within two hours after arrival using the disperser T18 Ultra Turrax with disposable dispersing elements (IKA-Werke, Staufen, Germany). Depending on the tissue structure, the speed range varied from 5,000 to 10,000 rpm for 30 s. For the bead milling method, Precellys Evolution homogenizer was used (Bertin Technologies, Rockville, Washington D.C., USA). The tubes were directly processed using two cycles at 7,200 rpm for 20 s each, interrupted by a pause of 20 s.

### Culture conditions

The homogenized periprosthetic tissue samples were applied for cultivation onto sheep-blood agar and chocolate agar (Oxoid, Basingstoke, Hampshire, United Kingdom). For anaerobic cultures, Schaedler agar, Schaedler KV agar (Oxoid, Basingstoke, Hampshire, United Kingdom) and Columbia blood agar (biomerieux, Marcy l’Etoile, France) were inoculated and incubated for five days. All specimens were also incubated for fourteen days using brain-heart infusion broth (BHI, Oxoid, Basingstoke, Hampshire, United Kingdom) and thioglycollate broth medium additionally incorporated with liver digest and finally supplemented with hemin and horse serum (LT, SIFIN, Berlin, Germany). For more details about this approach, see the literature [2, 3]. As an adjunct, the results of all investigated prostheses and components had no influence on the study and are therefore not discussed in more detail here.

The organisms were identified by Matrix-assisted laser desorption ionization-time of flight mass spectrometry (MALDI-TOF MS; BrukerDaltonics, Bremen, Germany) using the direct transfer method according to the recommendation of the manufacturer.

### Statistical analysis

The data for homogenization by disperser and bead milling were statistically analysed by the two-proportion z-test using the RStudio (version 1.2. 5042) software. Yate’s continuity correction was applied for all databases. *P* values of less than 0.05 should be considered statistically significant.

## Results

### Patient data

Ten cases were excluded because of an insufficient number of samples. Finally, a cohort of 80 patients, 40 with a hip and 40 with a knee prosthesis, was included in the study. In total, 722 tissue samples were investigated. In 35 cases the patients had at least one of the major diagnostic criteria for the presence of PJI (Table 1). Furthermore, five patients with no major criteria had an aggregate score of minor criteria greater than or equal to 6 and therefore were also considered as infected (Table 1). The other 40 cases had neither a major criterion for PJI nor an aggregate score greater than 2 and were classified as AF. For further demographic data, see Table 1.

**Table 1:** Clinical and microbiological characteristics of all study cases

	PJI^1^ (n = 40)	AF^2^ (n = 40)
Median patient age, yr (range)	75 (46–89)	72 (50–93)
No. (%) of females	18 (45)	24 (57)
Site of arthroplasty (no. [%])		
Hip	20 (50)	20 (50)
Knee	20 (50)	20 (50)
Type of surgery (no. [%])		
Explantation of the entire joint prosthesis	25 (62)	19 (47)
Explantation of prosthesis components (femoral, acetabular, tibial, inlay)	14 (35)	18 (45)
Debridement and prosthesis retention	1 (3)	3 (8)
Patients with major diagnostic criteria for PJI* (no./total no. [%])		
≥ 2 positive cultures	32/35 (91)	0
≥ 2 positive cultures + Presence of sinus tract	2/35 (6)	0
Negative culture + Presence of sinus tract	1/35 (3)	0
Average number of positive samples per patient/ Average total number of samples per patient	3.5/4.5	0/4.5
Patients with negative major criteria and minor pre- and intraoperative scoring based diagnostic criteria for PJI* (no./total no. [%])		
Score ≥ 6 (infected)	5/5 (100)	0
Score 4–5 (inconclusive)	0	0
Score ≤ 3 (not infected)	0	40

^1^Periprosthetic joint infection; ^2^Aseptic failure; *According to the 2018 Definition of periprosthetic hip and knee infection;

**Table 2A:** Culture results achieved by homogenization of tissue samples using Disperser vs. Bead milling method

PJI^1^ (n = 40)	Preference of method (no. [%])	Additional number of positive samples/ (No. of cases)
Culture positive (n = 34)	Concordance 23/(68)	0
	Disperser 7/(20)	1/(6); 2/(1)
	Bead milling 4/(12)	1/(1); 2/(3)
	Comments	Previously detected microorganisms
Culture negative (n = 6)	2 cases previously positive by TC*	*Cutibacterium spp.*
	4 cases previously positive by TC*	*Eikenella corrodens, Granulicatella adiacens, Actinomyces turicensis, Streptococcus* species., Coagulase-negative s*taphylococci* (CoNS)
AF^2^ (n = 40)		
Culture negative (n = 40)	Concordance 40/(100)	0

^1^Periprosthetic joint infection; ^2^Aseptic failure; *TC, tissue culture

### Microbiological diagnosis

In the PJI group, 34 patients yielded positive culture results. We processed a total of 308 tissue samples from these patients, 153 with the disperser and 155 with the bead mill. In the samples processed with the disperser 120 were positive. Processing with the bead mill yielded 121 positive results. On average, we received 4.5 samples per patient and method, of which 3.5 samples were positive (Tables 1 and 2B). However, in six cases only two samples were positive with both methods. In 23 patients (68%) we obtained identical culture results with both methods, in addition there was also a match in size, location and detected pathogens of the tissue samples. However, in 11 cases there were differences, but these were only related to the number of positive tissue samples per patient. In one of these cases, the tissue sample that gave a positive result when processed with the disperser was significantly larger than the sample processed with the bead mill. All pathogens were identified with both methods. The differences were distributed as follows: in six cases one sample and in one case two samples were additionally positive when the disperser was used (Table 2 A). On the other hand, in one case one sample and in three cases two samples were additionally positive when the bead mill was used (Table 2 A). Overall, there was no significant difference in the final evaluation, since in all cases at least two tissue samples were positive with both methods.

In the 34 positive cases a total of 51 microorganisms were recovered. The frequency of their occurrence is listed in Table 3. We identified 25 monomicrobial and nine polymicrobial infections. According to clinical records, 11 patients had a chronic course, in two of these cases small colony variants (SCV) were detected (Table 3).

**Table 2B:** The weight of each investigated tissue sample of all subjects and in case of PJI its effect on the rate of positive culture results using Disperser vs. Bead milling method

	Proportion of investigated samples based on weight			
PJI^1^ Culture positive (n = 34)	> 1.5 g	0.5-1.5 g	< 0.5 g	All sizes
Disperser (no. of positive cultures/ total no. of specimens [%])	71/87 (82)	41/53 (77)	8/13 (62)	120/153 (78)
Bead milling (no. of positive cultures/ total no. of specimens [%])	72/90 (80)	37/48 (77)	12/17 (71)	121/155 (78)
PJI^1^ Culture negative (n = 6)				
Disperser (no. [%])	18 (64)	7 (25)	3 (11)	27
Bead milling (no. [%])	15 (56)	7 (26)	5 (18)	27
AF^2^ (n = 40)				
Disperser (no. [%])	89 (49)	66 (37)	25 (14)	180
Bead milling (no. [%])	90 (50)	64 (36)	26 (14)	180

^1^Periprosthetic joint infection; ^2^Aseptic failure

**Table 3:** Microbiological findings by positive tissue cultures from 34 patients meeting the definition of PJI

Microorganisms	No. (%) (n = 51)
*Staphylococcus* species	19 (37)
*Staphylococcus aureus*	8 (16)
Staphylococcus epidermidis*	6 (12)
*Staphylococcus lugdunensis*	3 (6)
*Staphylococcus capitis*	2 (4)
*Enterococcus* species	5 (10)
*Enterococcus faecalis*	5 (10)
*Streptococcus* species	4 (8)
*Streptococcus dysgalactiae*	1 (2)
*Streptococcus agalactiae*	1 (2)
*Streptococcus constellatus*	1 (2)
*Streptococcus oralis*	1 (2)
Other Gram-positive cocci	2 (4)
*Parvimonas micra* (anaerobic)	1 (2)
*Peptoniphilus harei* (anaerobic)	1 (2)
Gram-positive bacilli	7 (14)
*Cutibacterium acnes* (anaerobic)	4 (8)
*Cutibacterium avidum* (anaerobic)	2 (4)
*Corynebacterium amycolatum*	1 (2)
Gram-negative bacilli	14 (28)
*Enterobacter cloacae*	4 (8)
*Escherichia coli*	3 (6)
*Klebsiella pneumoniae*	2 (4)
*Proteus mirabilis*	2 (4)
*Pseudomonas aeruginosa*	2 (4)
*Morganella morganii*	1 (2)

*in 2 cases associated with small colony variants (SCV)

Nevertheless, both methods yielded negative culture results for six patients in the PJI group. But all patients had been treated with antimicrobials because of microorganisms that had been detected previously from tissue specimens or synovial fluid (Table 2 A). In this group 54 tissue samples were processed, 27 with each method (Table 2B).

Specific analysis of the percentage of positive tissue samples based on weight revealed no significant difference between the methods. However, regardless of the method, the accuracy of the results decreased correspondingly to decreasing weight of the samples. Tissue samples with > 1.5 g had a positive rate of 82.0%, 71/87 for disperser versus 80.0%, 72/90 for bead milling, *P* = 0.79. For samples with a weight of 0.5-1.5 g we noted 77%, 41/53 for disperser versus 77%, 37/48 for bead milling, *P* = 0.97. And for samples with a weight of < 0.5 g we detected 62%, 8/13 for disperser versus 71%, 12/17 for bead milling, *P* = 0.60 (Table 2B).

In the AF group both techniques presented negative culture results in all 40 cases. Here, we processed 180 tissue samples with each method. For information on the weight distribution of the investigated tissue samples, see Table 2B.

A further aspect of our study was to record the origin of each specimen and its detection rate of microorganisms in the group of culture-positive PJI cases. Regardless of the joint, the highest rate of single samples was taken from the neo-synovium, followed by the acetabulum, if the hip was affected. In the number of samples taken from the proximal and distal periprosthetic membrane of the stem, there were joint-dependent differences.

Due to the low number of cases, we did not differentiate between surgical procedures. We cannot rule out that this had an influence on the different results.

**Fig. 1:**
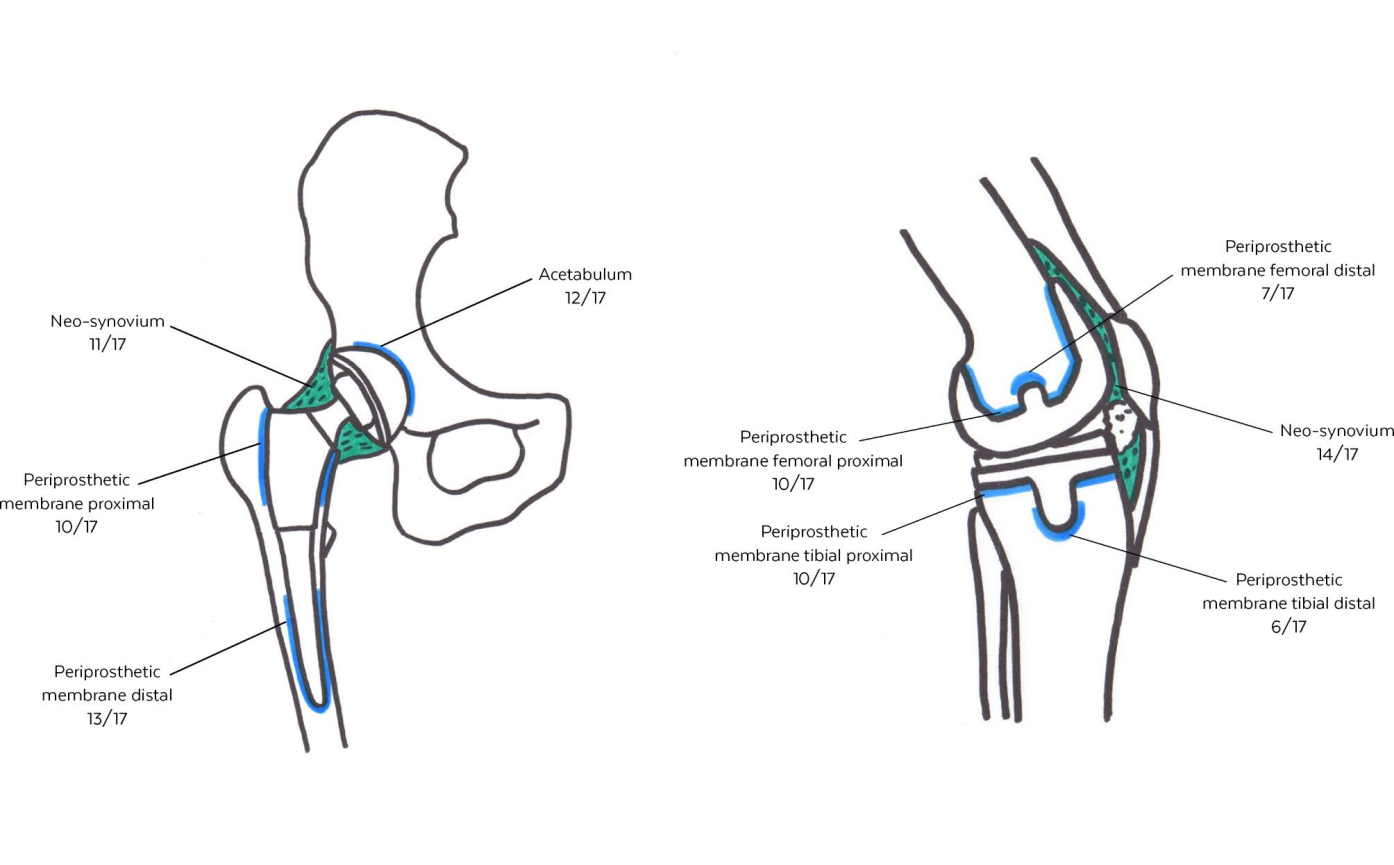
Overview of the local distribution of culture-positive tissue samples in patients with periprosthetic joint infection. Left: Hip (n = 17); Right: Knee (n = 17)

For an overview of the distribution of positive samples, see Fig. 1.

## Discussion

Correct identification of the causative agent from microbiological culture is mandatory for a targeted antimicrobial therapy of PJI. However, there is currently no consensus on several preanalytical and analytical aspects such as the most suitable tissue sample to be cultured, the optimal number of specimens investigated, the most effective method of tissue processing, and finally, the appropriate sensitive culture media that also enable detection of fastidious pathogens. We have already published research results on the latter [2, 3]. In this study we aimed to address the open questions in our patient collective.

Firstly, we recorded the origin of each tissue sample and its contribution to the detection of an infection (Fig. 1). In 25 of 34 culture-positive cases, samples from the neo-synovium were the best positive single location. The value especially of synovial biopsy in diagnostics of PJI both of the hip and knee was also reported by Fink et al. [7, 8].

The specifications in our study for both locations and volume are based on an evaluation of several thousand tissue samples that we have analysed over the past few years (not published).

One result of this investigation was that bone biopsies as a whole proved to be less suitable. This experience is confirmed by Larsen et al. who investigated, among other questions, the contribution of specimen types in the diagnosis of PJI [9].

Secondly, in our study, four tissue samples were sufficient to confirm the diagnosis of an infection. These results are in line with reports by Bemer et al. and Gandhi et al., who both demonstrated that four samples are optimal, if at least three different media including blood culture bottles (BCB) are used [10, 11]. We agree with the importance of culture media, however it is not the number but the composition of nutrients that matters. We would like to refer to our publications on this subject, especially with regard to the limited use of BCB [12].

Thirdly, although the patient samples were sent consecutively regardless of the course of symptoms, we were able to identify the pathogens of a series of chronic infections due to which the patients had had to live with prosthesis-associated pain for an average of 11 (5–25) months before surgery was carried out. Our processing thus enabled a targeted effective therapy for these cases. This can be regarded as an indication of the validity of both procedures, as smaller amounts of bacteria are generally expected with these types of infection.

Furthermore, in the AF group, neither procedure resulted in any contamination, and they therefore delivered an optimal specific result.

The literature on processing methods is rare. In 2017, Suren et al. published a prospective analysis of a semi-automated tissue homogenization method using the ULTRA-TURRAX drive workstation with tubes containing ten steel beads [13]. The authors investigated 38 total hip and knee arthroplasties, but their results were inconsistent, and no information was given about their routine procedures. Roux et al. published a retrospective analysis in 2011 [14] which included 92 patients undergoing revision surgery. The tissue samples were collected between 2003 and 2006 and examined using vials with added glass beads. The authors found a substantial number of microorganisms associated with PJI, but here too, they did not compare these data with their routine workflow. Redanz et al. first used an experimental model with a specimen of artificially inoculated pork to investigate the effectivity of Precellys Evolution bead mill homogenizer. The authors then processed clinical samples using 2 ml tubes and analysed 22 tissue samples from periprosthetic membranes and synovia recovered from seven patients. Only five samples were positive. Despite this limited amount of data, the authors gave a clear recommendation for the procedure [15]. It is worthy of note that according to the manufacturer, a loading capacity of up to 0.2 g weight is recommended for 2 ml tubes. In our experience, this volume is too small for a reliable diagnosis, especially if low-grade infections are suspected. In our study, about 90% of the samples had at least 5–10 times this weight. Only recently, Fang et al. demonstrated the superiority of tissue homogenization for diagnosis of PJI, but for comparison they used methods that have already proven to be non-competitive, such as manual techniques or pre-treatment of the tissue with ultrasound [16]. Finally, Yusuf et al. evaluated the diagnostic value of pre-processing tissue specimens with a homogenizer compared with their routine manual procedures. Surprisingly, the authors found no significant difference between the methods. Speculation remains as to whether the selected program was not suitable for processing these special tissue samples. The authors also did not provide any information as to the extent to which they carried out preliminary tests and why they selected the program mentioned in the material Sect. [17].

Even if we have demonstrated the accuracy of two homogenization techniques, our study has some limitations. Firstly, we accepted the bias that surgeons could assign the samples themselves, since transferring to another vial in the laboratory, even under laminar airflow, poses a risk of contamination. This free choice could be the reason why in seven cases processing under routine conditions revealed additional positive samples compared to processing by the bead milling method. But even with this method additional positive samples were found in four cases. However, these differences had no effect on the overall assessment. Secondly, there is no gold standard for processing procedures, making investigations very laborious, because all individual stages have to be carefully validated. In our study, we first had to establish which bead material (steel, glass or ceramics) was most suitable for our purposes. Then we had to identify both the appropriate mix of bead sizes for best homogenization and the right rotation speed without affecting the bacteria. Based on preliminary tests we decided on ceramic beads. The best ratio of homogenizing and recovery of bacteria was obtained at 7,200 rpm using a bead mix of 2.8/5.0 mm. However, at ≥ 8,000 rpm the temperature within the sample rose to 60° C and impaired bacterial growth.

Another aspect that has not yet been systematically investigated is the recording of the volume of the examined tissue samples [4]. Our monitoring showed a dependency between the volume and the detection rate of pathogens, but no difference in the methods used. However, if the weight was < 0.5 g, the bead milling method tended to achieve better results, although the proportion of positive tissue samples was the lowest overall (Table 2B). Even if the results were not significant, using this method might have a positive effect on preoperative arthroscopic diagnosis, especially of low-grade infections, as the volume of biopsies is often limited.

Independently of this finding, we are working on semi-quantitative PCR analyses to enable better integration of the informative power of molecular examination procedures into diagnostics in cases of unexpected culture-negative results (not finished).

## Conclusion

With this study we have demonstrated that two different semi-automated systems enable reliable processing of tissue samples for diagnosis of PJI. These techniques should replace the still widely used, less sensitive, manual methods which are more susceptible to contamination.

According to the literature, the performance and usability of the devices available in the market differ considerably. Therefore, comparative studies are urgently required. This study has also shown that the rate of positive tissue samples is influenced by the volume and exact origin of the sample. Therefore, these parameters should always be recorded and communicated in the laboratory report as they affect clinical relevance. There can be no doubt that standardized procedures are required to make the microbiological results predictable and comparable and give surgeons the highest level of certainty for their decision-making.

## Data Availability

The datasets generated and/or analysed during the current study are not publicly available due to protect individual privacy but are available from the corresponding author on reasonable request.
